# In vivo optimization of the experimental conditions for the non-invasive optical assessment of breast density

**DOI:** 10.1038/s41598-024-70099-x

**Published:** 2024-08-19

**Authors:** Nicola Serra, Rinaldo Cubeddu, Giulia Maffeis, Vamshi Damagatla, Antonio Pifferi, Paola Taroni

**Affiliations:** 1 Dipartimento di Fisica, Politecnico di Milano, Piazza Leonardo da Vinci 32, 20133 Milan, Italy; 2 Istituto di Fotonica e Nanotecnologie, Consiglio Nazionale delle Ricerche, Piazza Leonardo da Vinci 32, 20133 Milan, Italy

**Keywords:** Breast cancer, Cancer prevention, Cancer screening, Risk factors, Breast cancer, Cancer prevention, Cancer screening, Breast cancer, Cancer screening, Near-infrared spectroscopy, Applied optics

## Abstract

In this study, time domain diffuse optical spectroscopy is performed in the range 600–1100 nm on 11 healthy volunteers with a portable system for the quantitative characterization of breast tissue in terms of optical properties and optically-derived blood parameters, tissue constituent concentrations, and scattering parameters. A measurement protocol involving different geometries (reflectance and transmittance), subject’s positions (sitting and lying down), probing locations (outer, lower, and inner breast quadrants), and source-detector distances (2 and 3 cm) allowed us to investigate the effect of tissue heterogeneity and different measurement configurations on the results with the aim of identifying the best experimental conditions for the estimate of breast density (i.e., amount of fibro-glandular tissue in the breast) as a strong independent risk factor for breast cancer. Transmittance results, that in previous studies correlated strongly with mammographic density, are used as a reference for the initial test of the simpler and more comfortable reflectance measurement configuration. The higher source-detector distance, which probes deeper tissue, retrieves optical outcomes in agreement with higher average density tissue. Similarly, results on the outer quadrants indicate higher density than internal quadrants. These findings are coherent with breast anatomy since the concentration of dense fibro-glandular stroma is higher in deep tissue and towards the external portion of the breast, where the mammary gland is located. The dataset generated with this laboratory campaign is used to device an optimal measurement protocol for a future clinical trial, where optical results will be correlated with conventional mammographic density, allowing us to identify a subset of wavelengths and measurement configurations for an effective estimate of breast density. The final objective is the design of a simplified, compact and cost-effective optical device for a non-invasive, routine assessment of density-associated breast cancer risk.

## Introduction

Breast cancer is the neoplasia with the highest incidence and mortality among women and represents a significant strain for healthcare systems worldwide^1^. Early diagnosis increases therapeutical options and survival rates up to 99%^1^.

Except for gender and age, breast density is the most relevant risk factor involved in the development of breast cancer^2^. It is defined as the relative proportion between radio-opaque fibro-glandular tissue and transparent adipose tissue^3^. Nowadays breast density is estimated from the analysis of X-ray mammograms, which typically results in the assignment of the patient to one of the four BI-RADS categories^4^ (Breast Imaging Reporting and Data System), from category A, corresponding to the lowest density, to category D, corresponding to the highest density. Mammograms are most often acquired in the context of screening programs aimed at women not younger than 40 or 50 years old^5^, due to the ionizing nature of X-ray radiation and the lower sensitivity of mammography in the dense breast, more common in young women^6^. However, information on breast density could be useful earlier in life. It would allow one to implement at a younger age targeted monitoring of subjects with higher risk of cancer, develop personalized prevention paths and possibly lead to early cancer detection. From this picture, the pressing need for a non-invasive and effective way to assess breast density emerges, especially in young woman.

Diffuse optics (DO) investigates the absorption and scattering properties of tissue, which depend, respectively, on the chemical composition and on the size and density of scattering centers, that randomize the propagation direction of photons. This information can be used to distinguish fibro-glandular stroma from adipose tissue, or healthy tissue from cancerous lesions. Thus, diffuse optical methods can offer a non-invasive, compact, and cost-effective contribution to the detection and classification of breast lesions^7–20^, to therapy monitoring^12,21–30^ and to the estimation of breast density^31–33^. In the last decades, the application of DO to these aims has been extensively investigated by several research groups around the world, using different kinds of instrumentations, measurement protocols and data analysis methods^34–36^.

Lilge and coworkers used a broadband spectrometer and a halogen bulb as light source to assess breast tissue composition and found significant association between optically derived parameters and mammographic breast density^37,38^. Recently, they presented a modified version of the system, based on 12 continuous-wave (CW) diode lasers, finding that the reduction in the spectral information did not significantly affect the capability of the system to identify women with high-density breast^39,40^ and good results were obtained in terms of correlation between optical and X-ray derived data^32^. O’Sullivan et al.^22^ implemented a DO system that combines CW and frequency domain (FD) spectroscopy to monitor changes in breast density during neoadjuvant chemotherapy, obtaining significant correlations between optical parameters and Magnetic Resonance Imaging (MRI)-derived BI-RADS categories. Paulsen et al.^41^ developed an MRI-guided FD-DO tomographic system which makes use of 6 laser diodes in a relative narrow spectral range, from 660 to 850 nm, and of a photomultiplier tube detector. They demonstrated significant differences in water and total hemoglobin content and scattering parameters between in vivo fibro-glandular tissue versus in vivo adipose tissue. A more recent version of the system includes 3 more CW laser diodes, improving the spectral range up to 950 nm, and a hybrid detection array^42^.

We perform time domain Diffuse Optical Spectroscopy (TD-DOS) over a broad red and near-infrared spectral range (600–1100 nm) to retrieve estimates of breast tissue constituent concentrations (i.e., water, lipids, collagen), together with blood parameters (oxy- and deoxy-hemoglobin), that are informative on the metabolic properties of the breast, and scattering parameters (scattering amplitude *a* and power *b,* see section on “Data analysis”), which are associated with the microscopical structure of tissue (size and density of scattering centers, respectively).

Previously, a portable TD-DOS imaging system that performs a 2D transmittance scan of the compressed breast at 7 wavelengths (635–1060 nm) was used to perform breast density measurements on 146 subjects^31,43^. For each subject, four images were acquired (cranio-caudal and oblique of both breasts) and significant associations with mammographic BI-RADS categories were obtained for data averaged over the 4 images to provide the average breast tissue density of each subject^43^. In particular, we observed good positive correlation of water and collagen content, and negative correlation of lipids with BI-RADS categories. The scattering power *b* also showed a statistically significant increase in higher-risk categories (C and D). All relevant parameters were combined in an Optical Index (*OI*), which improved correlation with mammographic data. *OI* is defined as follow:

$$OI = \frac{{\left[ {water} \right]\left[ {collagen} \right]b}}{{\left[ {lipids} \right]}},$$

where square brackets indicate concentrations (mg/cm^3^).

A model based on logistic regression was also proposed with promising results for the identification of women at high risk using optically derived tissue parameters^31^.

Collagen Index (*CI*) is another synthetic parameter that showed strong correlation with mammographic density^44^:

$$CI = \left[ {collagen} \right] \cdot b$$

It combines collagen concentration with scattering properties, which, based on ex vivo measures^45^, seem to also depend on collagen. Thus, *CI* could be of great interest to assess what was hypothesized as independent collagen-related cancer risk^44^.

In a following study, the spatial information from the previous dataset was sub-sampled to test the feasibility of estimating breast density with just a few points measurement instead of a full scan of the breast^46^. The results showed that single-point measurements perform similarly and therefore a full map of the breast seems not necessary. This translates into a reduction of measurement times and of complexity and cost of the instrumentation.

These findings support the new approach chosen for the preliminary study we are reporting here and for the future clinical trial that is being planned. Instead of a high-spatial, low-spectral information acquisition, we moved to low-spatial, with just a few discrete measurements points on the breast, and high-spectral information acquisitions, using a broadband supercontinuum laser that provides a continuous emission spectrum from 600 to 1100 nm. In this laboratory study, measurements are performed on a few different positions to find the optimal location, or set of locations, for a reliable estimate of the overall average density of the breast. We use a transmittance geometry, with a mild compression of the breast, as in our previous studies mentioned above, and we also test a more patient-friendly configuration, in reflectance geometry, similar to clinical ultrasound measurements, which avoids the discomfort of compression for the patient and better suits the clinical workflow of a typical check-up routine.

Eleven healthy volunteers participated to the measurement campaign. The aims of the study are:

1. The optimization and validation of the portable TD-DOS system for in vivo measurements of breast density, in view of its future clinical implementation.
2. The identification of a subject’s and physician’s friendly measurement configuration in reflectance geometry (or more than one configuration) to be subsequently implemented in the clinical study.
3. The generation of an open dataset of in vivo data on the breast tissue, including broadband optical properties (absorption and reduced scattering coefficients), tissue composition (water, lipids, and collagen), blood parameters (total hemoglobin concentration and oxygen saturation), and scattering parameters (scattering amplitude and power providing) to investigate inter-subject and intra-subject variability.

The cohort of subjects involved in the study is relatively young (see Table 1 for demographics) and most of them are not of age for screening. For this reason, mammograms are not available for direct comparison of optical parameters with BIRADS categories. Transmittance acquisitions, that in previous were strongly correlated with mammographic breast density^43,46–48^, are used to validate the reflectance measurements in this preliminary study. In an upcoming clinical trial, recent mammograms will be available for the enrolled subjects, allowing us to correlate directly optical results from point-like, reflectance measurements with BI-RADS categories. The intent will then be to investigate the broadband optical properties of breast tissue and determine how much of this information is essential for a good estimate of breast composition and density, in order to identify a minimal set of wavelengths (and measurement points) that is sufficient for a good correlation with traditional mammography. The collection of this extensive set of measurements has involved a rather long measurement time of about 45 min (see “Measurement protocol” section). The study was approval by the Ethical Review Board of our Institution to be performed in our laboratory environment, where the length of the measurements is not a major issue. On the other hand, it was not feasible to enroll a broad number of subjects, all with recent X-ray mammograms available. The latter would only be feasible in clinical settings, but in that case a long measurement time would not be acceptable as it would not fit in the screening/diagnostic workflow. This has motivated our choice to separate the work in the initial small laboratory study followed by the broader clinical study.

**Table 1:** Age, BMI, and breast thickness (mm, from breast surface to chest wall, averaged over 3 locations on both breasts) of participants to the study.

Subject	1	2	3	4	5	6	7	8	9	10	11	Avg.	St.Dev.
Age (y)	60	69	25	25	35	30	25	38	48	35	44	39.5	14.0
BMI (kg/m^2^)	19.8	24.0	19.1	29.3	18.1	20.0	18.4	19.7	21.2	21.7	25.3	21.5	3.3
Thickness (mm)	36.3 ± 3.1	32.8 ± 3.9	27.8 ± 7.7	19.7 ± 3.1	18.3 ± 3.4	14.3 ± 2.4	12.8 ± 5.3	11.8 ± 1.9	11.5 ± 2.8	N.A	11.3 ± 3.2	19.7	9.8

Our final, long-term goal is the design and development of a small and cheap optical device for the estimation of breast density, operating at a few wavelengths in the range of 600–1100 nm, possibly in a comfortable reflectance geometry, sidestepping also the higher instrumental complexity of a transmittance acquisition. Such a device, being relatively cheap, simple, and completely harmless, could be easily integrated in a clinical workflow (for example, in gynecologists’ offices) and would allow for large-scale screening and routine check-ups with a quantitative approach, also in young women. This would allow one to develop personalized monitoring paths for subjects at high risk and implement personalized prevention interventions.

## Materials and methods

### Population study

Eleven healthy adult women were recruited to take part in the study. Demographic information (age, height and weight) was collected from participants and is reported in Table 1. The average age is 39 $\pm$ 14 and average Body Mass Index (BMI) is 21.51 $\pm$ 3.26 kg/m^2^. Table 1 also reports the average and standard deviation of breast thickness (distance from breast surface to chest wall) for each subject, averaged over 3 positions on both breasts, assessed using a B-mode ultrasound imaging system (see section “Measurement protocol”). Due to the young age of most participants, mammograms and BIRADS categories were not available.

This study received approval from the ethical committee of Politecnico di Milano and was conducted according to the ethical standards established by the Helsinki Declaration of 1975. Written informed consent was obtained from each subject enrolled in the study.

### System set-up

**Figure 1:**
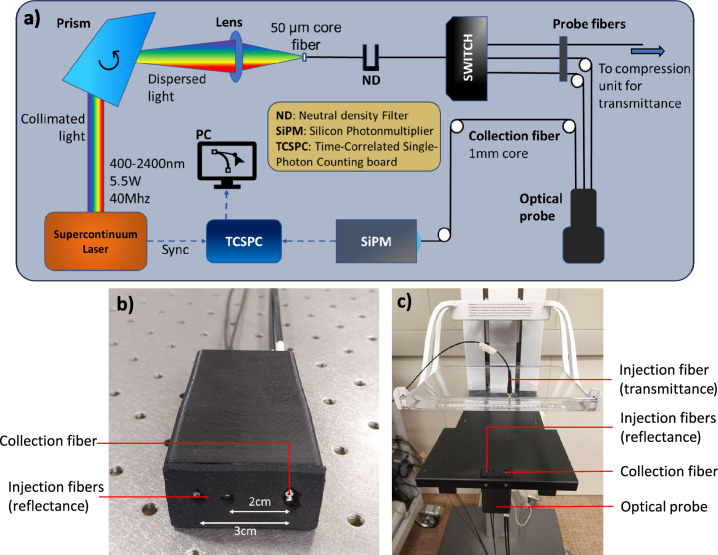
Layout of the experimental system (**a**), close-up of the optical probe (**b**), and close-up of the compression unit housing the optical probe (**c**).

Figure 1 presents the layout of the experimental system. A supercontinuum pulsed laser (SuperK Fianium FIU-15, NKT Photonics A/S, Denmark) is operated at a repetition rate of 40 MHz to generate a train of pulses with Full Width at Half Maximum (FWHM) of ~ 10 ps. Light is dispersed by an SF10 glass Pellin-Broca prism (Bernhard Halle Nachfl. GmbH, Germany) placed on a rotating stage, for wavelength selection in the spectral range of 600–1100 nm, at 10 nm steps. The radiation is coupled into a graded-index, 50-μm core diameter optical fiber. A computer-controlled variable neutral-density filter is used to attenuate and optimize the injection power. An optical switch allows for multi-distance and multi-configurational acquisitions, coupling the laser light into three different injection fibers with 100-μm core diameter. At the detection side, the light diffused from tissue is collected with a 1-mm core diameter step-index optical fiber and focused on a Silicon Photon Multiplier (SiPM) module with 1 mm^2^ active area (SiPM sensor: S10362-11-050C, Hamamatsu Photonics K.K., Japan), operated in single-photon regime. To reconstruct the temporal shape of the diffused optical pulses, the output signal of the detector is processed by an electronic board (SPC-130, Becker & Hickl GmbH, Germany) for Time-Correlated Single Photon Counting (TCSPC). The injection and collection fibers are housed in a probe (Fig. 1b) that can be either hand-held by an operator for reflectance measurements or placed in the lower plate of a motorized compression unit for both reflectance and transmittance acquisition (Fig. 1c, see Section on “Measurement protocol”). Wavelength selection, injection power optimization and probe-fiber switching procedures, together with data collection, are controlled and automated by in-house built software.

### Measurement protocol

The protocol comprises the acquisition of the Instrument Response Function (IRF), the measurement on a tissue phantom and, finally, in vivo measurements. Each acquisition corresponds to a spectrum (from 600 to 1100 nm, in steps of 10 nm) composed of 51 Distributions of Time-of-Flight (DTOFs) of the re-emitted (reflected or transmitted) light pulses and it takes about 90 s to complete. An integration time of 1 s is used to acquire each DTOF. To keep the measurement protocol relatively short, only one DTOF is collected at each wavelength and measurement configuration.

The IRF is acquired placing the injection fiber tip-to-tip with the collection fiber and using a thin layer of Teflon tape to excite all propagation modes. It allows us to check the system performance and account for system non-idealities when data are analyzed. Then, for reference and standard operation check, a measurement is performed on a resin phantom of known optical properties (absorption coefficient *μ*_a_ = 0.05 cm^−1^ and reduced scattering *μ*′_s_ = 10 cm^−1^ at 800 nm), mimicking tissue properties in the wavelength range of interest.

**Figure 2:**
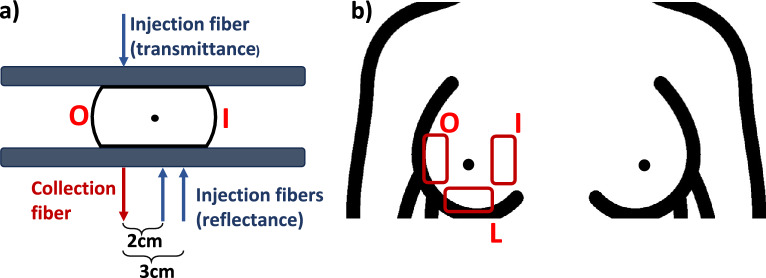
Measurement configuration on the right breast for a sitting subject with compressed breast (**a**) and for a supine subject (**b**). O, outer; L, lower; I, inner quadrants.

The in vivo protocol is divided into two measurement sessions, as schematized in Fig. 2 for the right breast, as an example. In the first session, measurements are performed with the subject sitting in front of a motorized compression unit, with the optical probe housed in the lower plate (see Fig. 1c). An operator helps the subject to correctly position the right breast on the plate (see Fig. 2a) and the first two spectra are acquired in reflectance geometry, respectively at 2 cm and 3 cm inter-fiber distance. Then, a mild compression is applied to the breast using the motorized compressor, up to a point where the subject is not feeling discomfort, and an acquisition in transmittance configuration is performed, in cranio-caudal view. The compression lasts about 90 s. The same sequence is repeated for the left breast. The breast is positioned on the optical probe, housed in the lower compression plate, so that the probed volume is centered across the breast midline for the reflectance measurement. This leaves the collection fiber slightly off-set with respect to the breast midline. Since the breast is not repositioned for the compressed transmittance acquisition, the actual location of this acquisition is displaced by 1–1.5 cm towards the outer quadrants for the right breast, and towards the inner quadrants for the left breast (as schematically presented in Fig. 2a for the right breast).

In the second session of the measurement protocol, the subject is asked to lay down supine on a clinical bed, similar to the position for a clinical ultrasound assessment. The operator holds the optical probe in place on the breast in three different positions (Fig. 2b) to investigate the effect of tissue heterogeneity. Starting from the right breast, the probe is firstly positioned between the outer quadrants of the breast (O), then between the lower quadrants (L) and finally between the inner quadrants (I). For each position, acquisitions are taken at 2 cm and 3 cm inter-fiber distance. The same procedure is repeated for the left breast.

Finally, 10 out of the 11 subjects also participated in a measurement session to assess by B-mode ultrasound imaging (E2 Exp., Sonoscape Medical Corp., China) the breast thickness (distance between skin and chest wall) in the same three positions (outer, lower and inner quadrants) as in the supine protocol, for both breasts. Subject #10 could not participate for medical reasons.

A complete measurement session on a single subject lasts about 45 min and yields a total of 18 spectra.

### Data analysis

The DTOF is fitted to a solution of the Diffusion Equation (DE) for a homogeneous semi-infinite medium (reflectance measurements) or an infinite slab (transmittance measurements) with the corresponding extrapolated boundary conditions suitable for the geometry of interest^49^. The theoretical model is convolved with the IRF to take into account the system non-idealities, such as the original pulse-shape of the laser source and the broadening and delay introduced by fiber propagation and detection system. The DTOF is fitted from 80% of the pulse peak on the leading edge to 5% on the falling edge.

Two different types of procedures are employed for data analysis, *i.e.* a standard fit and a spectrally-constrained fit. In the former, each of the 51 DTOFs corresponding to a different wavelength is fitted independently to the DE, using the absorption coefficient $\mu_{a}$ and the reduced scattering coefficient $\mu_{s}^{\prime }$ as free varying parameters. In the spectrally-constrained fit, all 51 DTOFs of a spectrum are fitted at once, to directly retrieve the tissue constituent concentrations and the scattering parameters, used as the free varying parameters^50^. The Lambert–Beer law for $\mu_{a}$ and an empirical power law for $\mu_{s}^{\prime }$ are replaced in the DE, obtaining not the optical properties of the tissue, but the concentrations of water, lipids, collagen, oxy- and deoxy- hemoglobin and the scattering amplitude *a* and power *b* of the medium. The Lambert–Beer law reads:

$$\mu_{a} \left( \lambda \right) = \mathop \sum \limits_{i} \in_{i} \left( \lambda \right) C_{i}$$

where $\in_{i}$ and $C_{i}$ are, respectively, the molar extinction coefficient and the concentration of the $i$-th constituent. Apart for the five constituents mentioned above, an extra concentration associated to a wavelength independent absorber with unitary extinction coefficient [*ϵ (λ)* = 1 cm^−1^] is also considered as a free varying parameter, to take into account potential additional chromophores that otherwise we might be ignoring. This additional fitting parameter does not significantly affect the retrieved concentrations of oxy-hemoglobin, water and lipids. Instead, it improves the decoupling of deoxy-hemoglobin and collagen contents, which have a rather unstructured absorption spectrum in the range of interest and a similar increase at low wavelengths. The background accounted on average for (0.018 ± 0.01) cm^−1^.

The empirical power law for the reduced scattering coefficient comes from the Mie theory of scattering and reads^51,52^:

1 $$\mu_{s}^{\prime } \left( \lambda \right) = a \left( {\frac{\lambda }{{\lambda_{0} }}} \right)^{ - b}$$

where ${\lambda }_{0}$ = 600 nm is a reference wavelength and $a$ and $b$ are called, respectively, scattering amplitude and power and are associated, respectively, to the density and size of scattering centers.

### Statistical analysis

The linear correlations among tissue components and scattering parameters are investigated through the Pearson linear correlation coefficient *r*. The same test was used to analyze the correlation between results from different measurement configurations (*e.g.*, outer quadrants *vs* inner quadrant, or *ρ* = 2 cm *vs ρ* = 3 cm), while the significance of their difference is assessed using the Wilcoxon matched-pairs signed-rank test. Bonferroni correction is applied to adjust p-values for multiple testing^53^. This correction might be excessively strict since a relatively small sample size is involved and the tests we consider are expected to be not completely independent. Therefore, when statistical significance is lost due to Bonferroni multiplicity adjustment, the p-value before adjustment is also reported. Tests are applied with a level of significance *α* = 0.05 and results are considered statistically significant when *p* < 0.05.

## Results and discussion

### Optical properties

**Figure 3:**
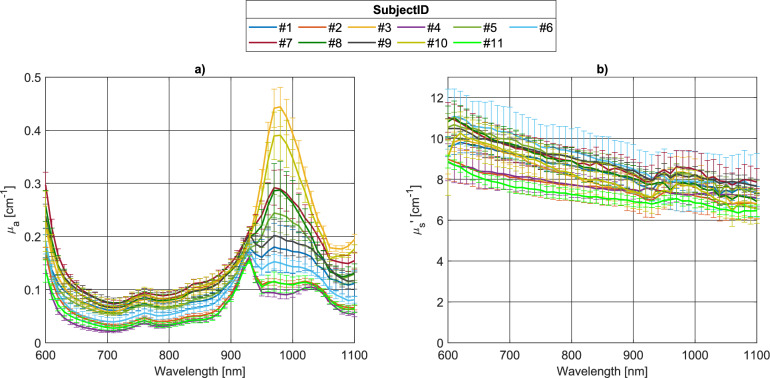
Absorption (**a**) and reduced scattering (**b**) spectra of the 11 subjects (averaged over left and right breasts, and all measurement locations on the breast), in supine, reflectance configuration, at $\uprho$  = 3 cm.

To provide a general overview of the inter-subject variability of the optical properties of the breast in reflectance geometry, Fig. 3 shows the absorption and reduced scattering spectra of the 11 subjects, averaged over left and right breasts and the three different measurement locations on the breast (outer, lower and inner quadrants) in the supine-subject configuration, at inter-fiber distance $\uprho =$ 3 cm.

The strong inter-subject variability in terms of tissue composition (and therefore, density) is reflected by the optical properties, especially by the absorption coefficient at wavelength $\lambda >$ 900 nm, where the absorption peaks of lipids (930 nm) and water (970 nm) (*i.e.*, the major breast constituents) are localized. The differences in the absorption coefficient around 1100 nm could be associated with different collagen contents. Also, at short wavelengths, where the concentration of hemoglobin has the greatest impact, a large variability in absolute absorption is observed. Subjects with higher absorption contribution from water (*i.e.*, #3, #7, #8, #10), and presumably also collagen, are characterized by a higher absorption on the whole spectrum, also at lower wavelengths, indicating a higher concentration of hemoglobin; while subjects with a predominant lipid peak (*i.e.*, #2, #4, #6 and #11) exhibit a lower absorption spectrum at all wavelengths.

The strong absorption (*µ*_a_ > 0.4 cm^−1^) near the water peak for some of the subjects might represent an issue in terms of signal-to-noise ratio (SNR, as it was the case in these measurements for some transmittance acquisitions, where the signal attenuation is higher due to longer source-detector distance) to be considered in the design of the dedicated system. In a previous study^54^, we demonstrated signal level compatible with transmittance acquisitions that are effective to estimate breast density, exploiting an array of 8 SiPMs. In the present study, a single SiPM was used, but it can easily be replaced by the same probe as used in the other system to provide higher signal level. The high absorption might also reduce the accuracy of the diffusive model, since one of its assumptions is that light propagation in the medium is isotropic, which is satisfied when the absorption coefficient is negligible with respect to the scattering coefficient^49^. The limit of the model becomes apparent for the reduced scattering coefficient (Fig. 3b) at long wavelengths, where it deviates from the power decay described by Eq. (1) due to a significant coupling with the absorption coefficient. However, the scattering estimate is still reliable in the first part of the spectrum (600–900 nm), which is sufficient for fitting to the power law and effectively retrieving scattering amplitude and power. If we focus our attention on this part of the scattering spectra, we note that flatter curves are associated to subjects with adipose breast (*i.e.*, #2, #4, and #11), as expected for lipid-dominated tissue. This is confirmed by the negative Pearson linear correlation coefficient between lipids concentration and the scattering power *b* (*r* = − 0.966, *p* = 8.1 × 10^–5^).

Similar trends of the inter-subject variability of the absorption and reduced scattering properties are obtained also for $\rho =$ 2 cm (Fig. S1 in the Supplementary Material), even though, especially for dense breasts (Subjects #3 and #10), where the absorption spectrum is dominated by water absorption around 970 nm, an overall lower absorption is estimated at that wavelength, in agreement with lower sensitivity to the deep fibro-glandular tissue.

**Figure 4:**
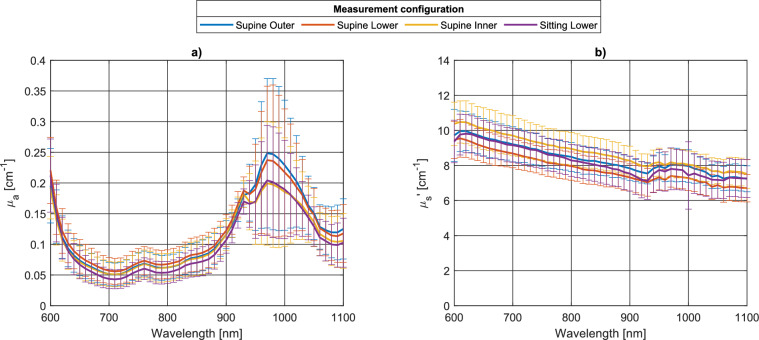
Absorption (**a**) and reduced scattering (**b**) spectra (average and standard deviation over left and right breasts of the 11 subjects) in reflectance at ρ = 3 cm, subject in supine position (outer quadrants, blue; lower, red; inner, yellow) and sitting (violet).

Figure 4 plots the absorption (a) and reduced scattering (b) spectra averaged over both breasts and all subjects, for the reflectance measurements at inter-fiber distance $\rho =$ 3 cm, in supine position on the outer, lower, and inner quadrants, and the reflectance measurements on the lower quadrants in the sitting position, showing how, on average, the optical properties change with measurement position on the breast. The outer quadrants probe a stronger water absorption, consistent with breast anatomy since the mammary gland is mostly located in the outer portion of the breast. Measurements on the lower quadrants in the supine configuration retrieve a more marked water peak (at 970 nm) than in the sitting configuration, in agreement with the fact that the superficial adipose tissue is flattened, due to the subject’s position, and allows a deeper probing of the underlying fibro-glandular water-rich tissue. No clear dependence of the average scattering properties on measurement position/configuration is observed.

Beyond the average trend of the absorption properties, the spectral changes in the standard deviation highlight in a synthetic way how much the absorption properties are affected by the different tissue composition of individual subjects (from adipose breasts, dominated by lipid absorption at 930 nm to high density breasts, with remarkable water absorption at 970 nm. Due to the strong water absorption around 970 nm, differences in its concentration due to different breast types lead to a marked inter-subject variability in this spectral range.

Figures representing data for some individual subjects are presented in the Supplementary Material. Supplementary Figs. S2, S3 and S4 display all reflectance measurements at $\rho =$ 3 cm for Subject #4, who has generally low absorption and a clear lipidic peak at λ ≈ 930 nm, compatible with an adipose breast; Subject #10, who has overall high absorption, attributed to blood, and a marked water peak at λ ≈ 970 nm, suggesting a dense breast; and Subject #8, who presents intermediate spectral features between the previous cases, as expected for a mixed breast. This representation suggests that increasing amounts of fibro-glandular tissue correspond to more marked variations of the optical properties with the probing position on the breast. This is especially evident for the absorption properties at *λ* > 900 nm, attributed to strong water absorption, but holds also for short wavelengths, mostly dominated by blood absorption. Some of the subjects (#1 and # 5) also show a substantial change in spectral features among the different probing positions. That occurs especially with mixed type breasts, which show higher heterogeneity than clearly adipose or fibro-glandular breasts.

### Tissue composition

**Figure 5:**
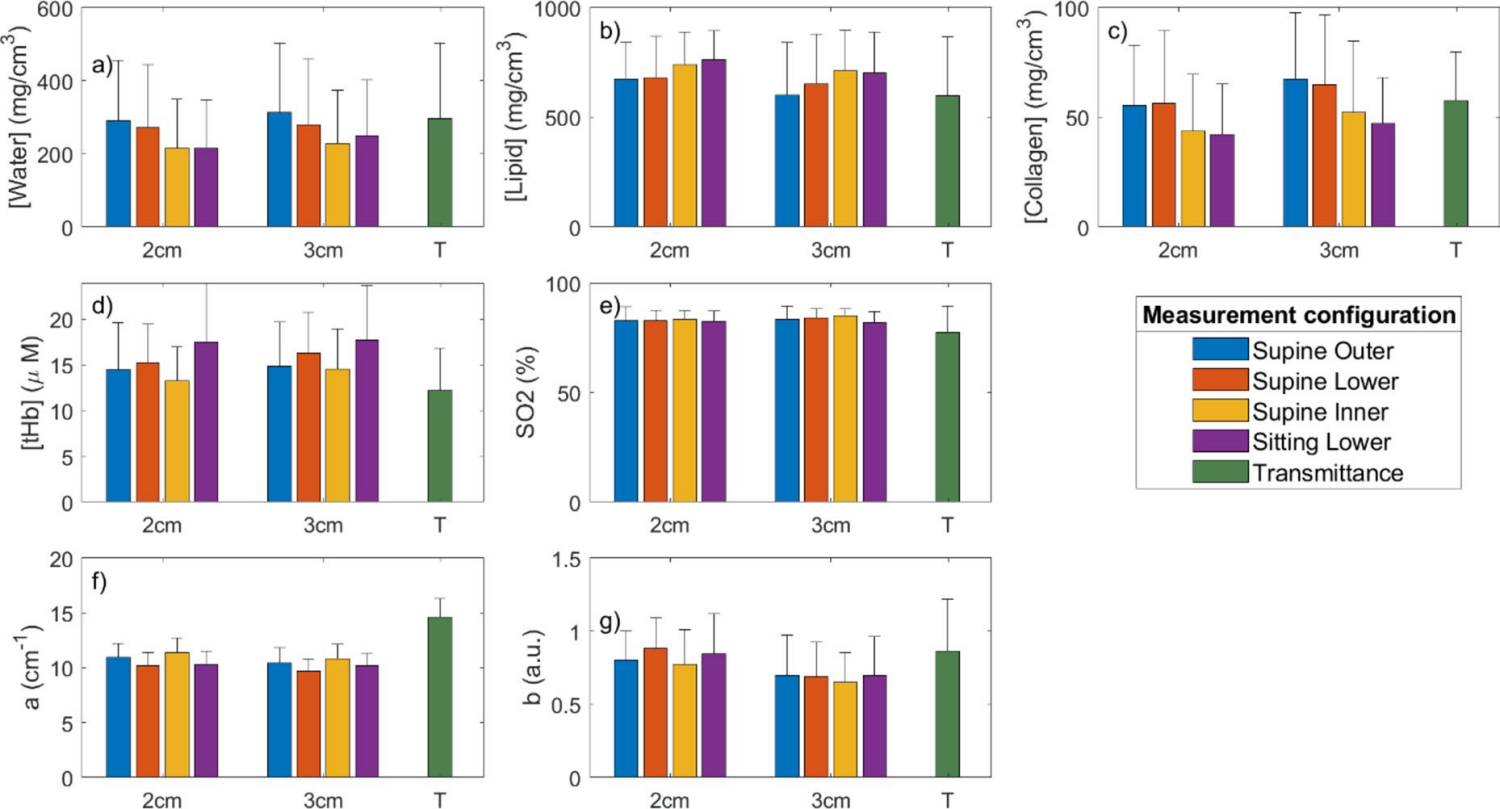
Averages and standard deviations over all subjects and both breasts of tissue composition (**a**–**c**), blood parameters (**d**, **e**) and scattering parameters (**f**, **g**) obtained from reflectance measurements on supine subjects (outer, lower, and inner quadrants) and sitting subjects, with ρ = 2 cm and ρ  = 3 cm (left and middle group, respectively), and from transmittance acquisitions (T, bar on the right).

As described in the section “Data analysis”, the DTOFs are analyzed with a spectrally-constrained method to directly retrieve an estimate of the main tissue constituent concentrations (water, lipid and collagen), blood parameters (oxy- and deoxy-hemoglobin, then used to derive total hemoglobin *tHb* and oxygen saturation *SO*_*2*_) and scattering properties (amplitude *a* and power *b*). Figure 5 presents these parameters, averaged over all subjects and both breasts, for $\rho =$ 2 cm and $\rho =$ 3 cm inter-fiber distances and for the same 4 configurations as in Fig. 4. As a link to previous results that showed the effectiveness of transmittance acquisitions for the estimate of breast density^43,46–48^, transmittance data obtained in the present study are also included in Fig. 4. Table 2 reports the linear correlation coefficients and relative *p*-values between transmittance results and the different reflectance results, in terms of the seven optically derived tissue parameters.

**Table 2:** Correlation coefficients (r) and p-values (p, adjusted for multiple testing) for the tissue constituent concentrations and scattering parameters retrieved in the transmittance acquisition against reflectance acquisitions.

	Sitting				Supine			
	2 cm		3 cm		2 cm		3 cm	
	r	*p*	r	*p*	r	*p*	r	*p*
tHb	0.656	0.004**	0.724	0.001**	0.609	0.011**	0.598	0.013**
SO_2_	0.125	1.0	0.341	0.481	0.282	0.813	0.164	1.0
Lipids	0.877	3.5E−07**	0.919	5.9E−09**	0.908	2.1E−08**	0.966	1.4E−12**
Water	0.906	2.5E−08**	0.924	3.5E−09**	0.968	6.7E−13**	0.977	2.8E−14**
Collagen	0.300	0.701	0.507	0.064*	0.802	2.9E−05**	0.750	2.3E−04**
*a*	0.604	0.012**	0.511	0.060*	0.515	0.056*	0.488	0.084*
*b*	0.807	2.3E−05**	0.796	3.7E−05**	0.766	1.3E−04**	0.792	4.4E−05**

**p-value significant after Bonferroni adjustment; *p-value significant only before Bonferroni adjustment.

For all parameters, values derived from transmittance acquisitions are within the range of previous data obtained on a wider population with the transmittance imaging system, that showed good correlation with BIRADS categories^46^. At the same time, transmittance results correlate with supine reflectance results (see Table 2) for all parameters of interest for breast density (water, lipids, collagen, tHb, and *b*). Correlation with sitting reflectance results is significant for water, lipids, tHb and b, but weak for collagen. In particular, at *ρ* = 2 cm no significant correlation is found for collagen. At *ρ* = 3 cm, the correlation is significant before correcting for multiplicity (p = 0.016), but it is only close to significance after Bonferroni correction (p = 0.064). It is worth noting that Bonferroni correction might be excessively strict for these tests, because they are not independent (since concentrations of different chromophores in different quadrants are correlated) and we are testing on a relatively small sample (*N* = 11). Total hemoglobin (tHb) and oxygen saturation (SO_2_) are noticeably lower in transmittance results. These trends could be caused by the different volume probed in transmittance geometry, but could also be due to a lower blood perfusion in the breast due to compression during transmittance measurements. Indeed, reduction in breast blood volume and perfusion due to compression were previously observed by other investigators^33,55–57^. SO_2_ derived from reflectance measurements is not correlated with transmittance results, but it is worth mentioning that correlation coefficients and p-values improve and become significant when subjects with low SNR in the transmittance acquisition (Subjects #7 and #10) are removed from the analysis (in the latter case, before multiplicity correction, for Sitting—2 cm: r = 0.496, *p* = 0.036; Sitting—3 cm: r = 0.508, *p* = 0.032; Supine—2 cm: r = 0.570, *p* = 0.014; Supine—3 cm: r = 0.535, *p* = 0.022). The scattering amplitude *a* is significantly correlated only before multiplicity correction (except for the sitting position at *ρ* = 2 cm, which remains significant also after correction). It is worth observing that values are noticeably higher in transmittance. This is coherent with previous findings on a different cohort of subjects, where a similar sitting measurement protocol was performed with a different time domain diffuse optics instrument^58^. The correlation between transmittance and reflectance results, and particularly with supine-subject measurements, in terms of the most relevant optical parameters for the estimate of breast density (water, lipids, collagen, tHb, and *b*) provides an initial support to the new reflectance, single-point acquisition approach. In the following extensive clinical trial, reflectance results will be directly tested against radiological BIRADS categories to confirm the quality of the results obtained with this protocol.

Focusing on the dependence of reflectance outcomes on the probed region of the breast, a progressive increase in adipose tissue (lipids) and decrease in fibro-glandular stroma (water and collagen) can be observed moving from the outer to the lower to the inner quadrants in the supine subject configuration, as expected (*e.g.*, at *ρ* = 3 cm, Lipid [mg/cm^3^]: 602 ± 238 outer, *vs* 653 ± 227 lower, *vs* 711 ± 188 inner. Water [mg/cm^3^]: 314 ± 189 outer, *vs* 279 ± 180 lower, *vs* 228 ± 145 inner. Collagen [mg/cm^3^]: 67.2 ± 30.5 outer, *vs* 64.8 ± 32.0 lower, *vs* 52.2 ± 32.6 inner). Only the trend of collagen at ρ = 2 cm is less evident, possibly due to the difficulty in accurately estimating collagen, which is a much weaker absorber than water and lipids. Standard deviations are high because of the strong inter-subject variability, previously discussed in terms of optical properties (section “Optical properties”), which is reflected in the variability of the concentration of tissue constituents and scattering parameters. A visualization of this variability is given in Fig. S5 in the Supplementary Material, where tissue parameters are presented independently for each subject. However, it is important to note that the difference between inner and outer quadrants assessed with the Wilcoxon paired signed-rank test is statistically significant for water and lipid concentrations (Water *p* = 0.015; Lipid *p* = 0.029) and close to significance for collagen concentration (*p* = 0.056, but it is significant before Bonferroni adjustment: *p* = 0.019). As noted in the previous section on “Optical properties”, this trend is coherent with the anatomy of the breast, since the mammary gland, composed of fibro-glandular stroma, rich in water and collagen, is located in the outer portion of the breast^59^. Measurements in the lower quadrants yield intermediary values, as this probing position is prone to include contributions from both the outer and inner quadrants.

Total hemoglobin is lower in the inner quadrants with respect to lower quadrants (*p* = 0.015), while no trend is reported when comparing outer quadrants with the others. No significant trend is observed in oxygen saturation and scattering power b from outer to lower, to inner quadrants. Scattering amplitude *a* is higher in the inner quadrants with respect to lower quadrants (*p* = 0.015).

Measurements at *ρ* = 3 cm consistently result in higher density tissue (lower lipids and higher water and collagen) than at *ρ*  = 2 cm, suggesting better probing of deep tissue, since the fibro-glandular stroma is concentrated deeply in the tissue, covered by a subcutaneous adipose layer (averaging over all measurements, Lipid [mg/cm^3^]: 713 ± 166 at 2 cm, *vs* 666 ± 216 at 3 cm. Water [mg/cm^3^]: 248 ± 155 at 2 cm, *vs* 267 ± 171 at 3 cm. Collagen [mg/cm^3^]: 49.4 ± 28.4 at 2 cm, *vs* 57.8 ± 30.6 at 3 cm). This difference is confirmed by the Wilcoxon test between the two inter-fiber distances for the three main tissue constituents (Lipid *p* = 0.019; Water *p* = 0.010; Collagen *p* = 0.010). The scattering power *b* seems to be significantly lower at 3 cm (*p* = 0.001). This trend was already detected in a previous study with similar measures at different inter-fiber distances in the sitting position^58^.

Measurements in the sitting-subject configuration in reflectance geometry seem to retrieve more adipose tissue (more lipids, less water and collagen) than in the supine subject on the inferior and external quadrants, while concentrations are similar to what is obtained on the internal quadrants of the supine subject. This is in line with what was observed for the absorption spectra and may derive from breast flattening of the supine subject that leads to thinner subcutaneous adipose tissue layer. Sitting acquisitions also results in marked higher hemoglobin content than any supine measurement. This trend might be related to changes in blood circulation when lying down, coherently with what has been observed by different studies that monitored blood parameters for different phlebotomy postures when lying down, sitting, and standing^60,61^.

**Figure 6:**
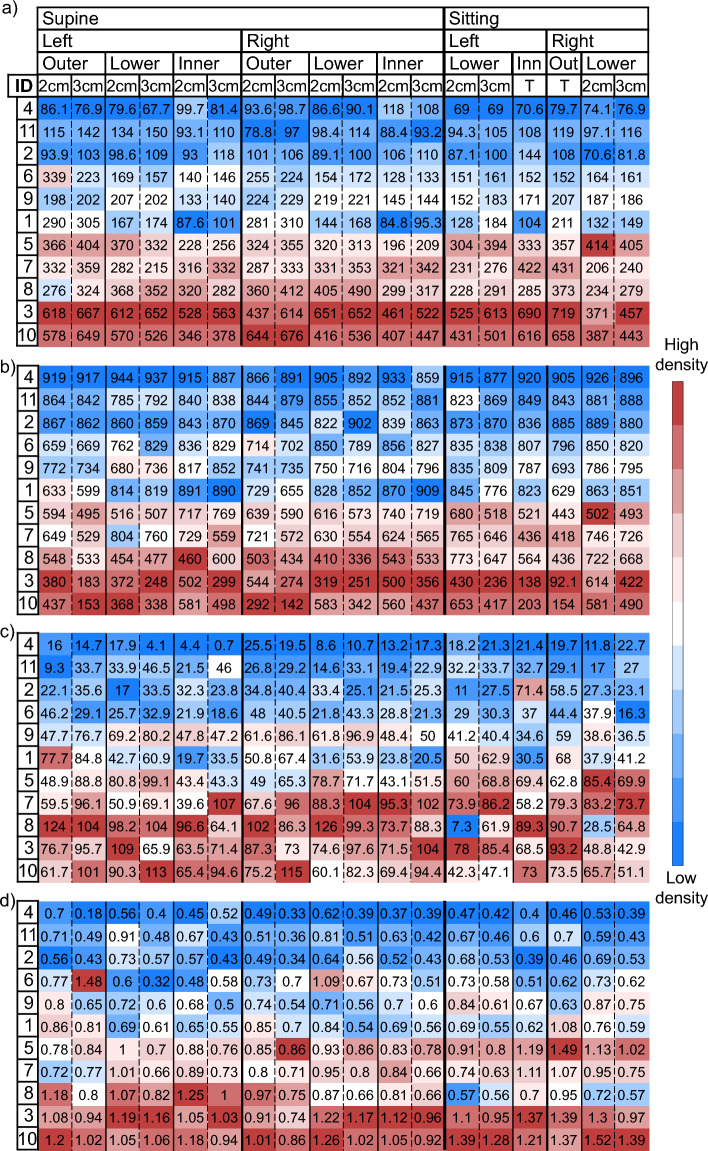

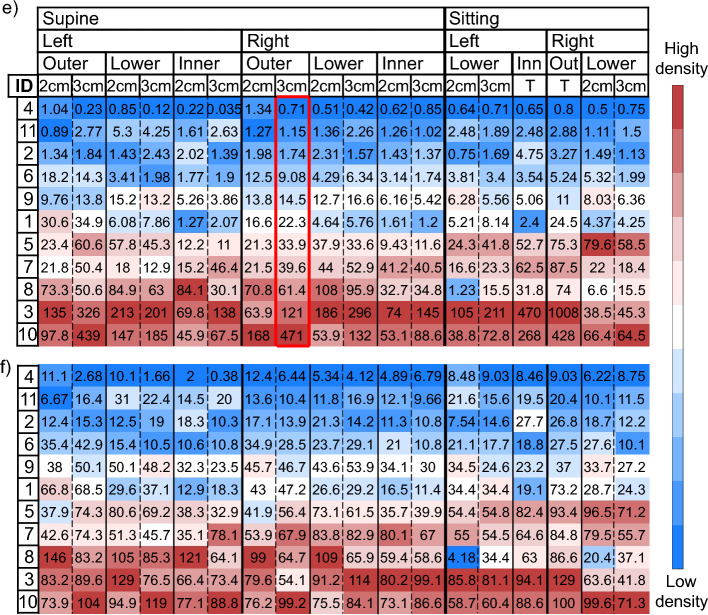
(**a**) Water, (**b**) lipid, (**c**) collagen, (**d**) scattering power b, (**e**) OI, and (**f**) CI for each subject (on rows) as a function of subject position (supine, sitting), breast (left, right), probing position (outer, lower, inner quadrants) and source-detection distance (2 cm, 3 cm, T = Transmittance). Values are color-coded from lower (blue) to higher (red) density within each column. The column with red margins was used as a reference for ordering all subjects.

In the supine protocol, the injection and collection fibers are orthogonal to the chest wall and data collected to characterize breast tissue could be potentially affected by the underlying chest muscle, which is rich in water and blood and have generally high absorption. Other research groups have investigated with simulations the effect of the chest muscle tissue on frequency domain diffuse optical tomography of the breast, showing it can affect the reconstructions significantly^62,63^. However, our study is performed in different experimental conditions (time domain data collected with source-detector distance not higher than 3 cm and interpreted with a homogenous model). Thus, the effect of the chest wall could also be different. In a phantom study using similar experimental conditions (time-resolved diffuse reflectance spectroscopy) we demonstrated that whenever the upper layer is less absorbing than the lower layer—as in the case of breast overlying the chest wall—the sensitivity to deep structures is reduced as compared to the opposite case^64^. Besides this general property that reduces the risk of contamination, we assessed the distance between skin and chest wall using B-mode ultrasound imaging in the same three positions as investigated with diffuse optics. Average and standard deviations for each subject are reported in Table 1. Subjects with the shallowest chest wall are #6, #9, and #1, with a distance just above 1 cm (respectively, 11.3 mm, 11.5 mm, and 11.8 mm). However, they do not have the highest absorption spectra nor water and tHb concentrations (Fig. 3a and Fig. S5 in the Supplementary Material), which would be expected if the chest muscle tissue contribution played a significant role in the estimated optical properties. The correlation between optical parameters and breast thickness (Table S1 of the Supplementary material) is statistically significant but weak for most optical properties (correlation coefficients range from r = 0.3 to r = 0.45, except for *a* that reaches r = 0.67). However, these correlations are not necessarily attributable to the chest muscle contribution, since a large breast is often richer in adipose tissue than a small one. Finally, measurements performed in the sitting position, with the probe parallel to and far from the chest, are not expected to be significantly affected by its contribution. Results obtained from reflectance measurements in supine position show good correlation with the corresponding transmittance measurements on the sitting subject (Table 2). Moreover, the supine reflectance configuration retrieves optical estimates that are in agreement in absolute values (see Fig. S5 in Supplementary Material), and in the ranking of subjects (Fig. 6 in the following section) with the other geometries. This again indicates that the chest muscle tissue does not play a significant role in our characterization of the breast tissue and that the supine reflectance measurement configuration is adequate to estimate breast composition.

### Estimate of breast density

One of the aims of this study is to check which tissue constituents, parameters and/or measurement configuration can provide the most effective information for estimating breast density. To this end, Fig. 6 shows results in terms of: (a) Water, (b) Lipids, (c) Collagen, (d) Scattering power, (e) Optical Index, and (f) Collagen Index for each subject as a function of subject position (supine, sitting), breast (left, right), probing position (outer, lower, inner) and source-detection distance (2 cm, 3 cm), and also for transmittance acquisitions (T). Subjects are listed in ascending order according to *OI* values as estimated on the supine subject, right breast, outer quadrants at 3 cm inter-fiber distance (this configuration was chosen arbitrarily, but, as evident from the figure, choosing a different configuration to order data would not change the outcomes significantly). For each measurement configuration (*i.e.*, each column), results for all subjects are color-coded from lowest (dark blue) to highest (dark red) values for all optical parameters that, based on breast physiology and previous optical assessments^43,46–48^, are expected to increase with mammographic breast density (i.e., water, collagen, scattering power *b*, OI, and CI), while the opposite color-coding is used for lipid content, which is negatively correlated with breast density. Since recent mammograms are not available for the enrolled subjects, the direct investigation of the correlation of optical parameters derived in different measurement configurations with breast density is not possible. Therefore, the approach adopted here is to investigate whether the optical estimates obtained in the different measurement conditions yield compatible rankings of the subjects and if they agree with what obtained previously on a wider cohort of subjects in transmittance geometry, where the OI was used for breast density ranking^46^.

From such representation we note a generally consistent color gradient among all measurement configurations and parameters, indicating that generally they retrieve a consistent density classification for the subjects involved in the study. In particular, transmittance results are in good agreement with the ranking of subjects obtained in the different reflectance configurations, suggesting that the latter acquisitions might be able to derive an effective estimate of breast density, as transmittance geometry proved to be in previous measurement campaigns. The color gradient is more uniform in water and lipids than in collagen estimates. This is because collagen quantification can be less accurate, due to the overall weak absorption of collagen and to the overlap of its absorption line shape with that of the strongly absorbing deoxyhemoglobin at short wavelengths and with that of lipids and water at long wavelength^44^. However, information on collagen content is of great interest since it is directly involved in the development of breast cancer and is itself a potential risk factor^65–67^ Previous in vivo and ex vivo studies^45^ have found that the scattering power *b* correlates with collagen and this is confirmed by our results (*r* = 0.844, *p* = 0.007). Thus, the Collagen Index (*CI*), that combines collagen concentration with the scattering power *b*, enhancing their correlation, could amplify their informative content. The Optical Index (*OI*) combines all tissue constituents contributing to mammographic density and could potentially provide a stronger estimate of breast density^43^. Extreme cases, corresponding to subjects in the first positions (subjects #2, #4, and #11) and last positions (#3, #8, and #10) of the ranking are easier to identify and most parameters and measurement conditions agree on the classification. This is important since the main aim of a measurement of density is to distinguish between groups of women with different risk levels.

This color-coded representation confirms that, even if the estimated values are different, 2 and 3 cm are comparable for ranking water, lipids, collagen, and scattering power* b* (Pearson correlation coefficients: *r* = 0.997, *r* = 0.98, *r* = 0.96, and *r* = 0.97, respectively). Very strong correlation between data obtained at different inter-fiber distance is obtained also combining parameters in a synthetic index (*r* = 0.95 for *OI* and average *r* = 0.96 for *CI*). From these results we can infer that a single source-detector distance is enough to estimate breast density. Moreover, since the objective is to measure the overall density of the breast, the highest source-detector distance allowed by the signal level should be chosen, since it ensures the deepest photon penetration and the largest probed volume.

Measurements performed on left and right breasts are well correlated (*r* = 0.98 for water, *r* = 0.98 for lipids, *r* = 0.96 for collagen, *r* = 0.95 for *b*, *r* = 0.97 for *OI,* and *r* = 0.98 for *CI*). This could suggest that measurements on a single breast are sufficient for an estimate of the overall density. However, previous studies found breast density asymmetry to be higher in women who develop breast cancer, and it was proposed as a risk factor itself^68,69^. For this reason, it is worth to keep performing measurements on both breasts.

## Conclusions

In this work, broadband TD-DOS from 600 to 1100 nm was used to conduct a thorough characterization of breast tissue in terms of optical parameters (absorption and reduced scattering coefficient), composition (water, lipids, collagen concentrations), blood parameters (total hemoglobin and oxygen saturation), and microscopical structure (scattering amplitude *a* and power *b*), highlighting the variability in these parameters among the 11 subjects recruited for this preliminary study. We investigated the effects of tissue heterogeneity (three different positions on the breast, on both breasts) and different measurement configurations (supine *vs* sitting subject, reflectance *vs* transmittance, 2 *vs* 3 cm source-detector distance) on the results. The Wilcoxon paired test highlighted the significant difference in the absolute values of some parameters between specific measurement configurations (*e.g.*, water, lipid, and collagen content in the outer quadrants *vs* inner quadrants, or in *ρ* = 2 cm *vs ρ* = 3 cm), proving the sensitivity of the technique to the physiology of the breast. Anyway, different configurations generally give comparable results in terms of estimate of breast density and risk classification, thus underlying the quality of the results.

The dataset built thanks to this laboratory study serves as a reference to devise an effective measurement protocol for the following extended clinical trial that will include a broader population. This protocol should be streamlined and convenient enough to be integrated seamlessly into the clinical workflow, yet it must encompass all necessary measurement configurations to attain the most comprehensive information regarding breast tissue composition and structure. For these reasons, only the supine-subject configuration will be kept. The exclusion of measurements in the sitting configuration, including transmittance measurements, removes the need for the compression unit, simplifying noticeably the implementation of the clinical system and the measurement protocol, since compression of the breast will not be needed. Furthermore, it should be considered that it is more comfortable for the patient. Also, measurements will be performed at 3 cm inter-fiber distance, to investigate the largest possible volume with a sufficient SNR. Measurements on the two breasts yielded similar results. However, it should be considered that asymmetry in breast composition was observed in previous studies and suggested as a potential risk factor^68,69^. Therefore, in the upcoming study it is still worth investigating both breasts. Finally, at least two (outer and inner quadrants) of the three probing positions on the breast will be reproposed to further investigate the effects of tissue heterogeneity also in a cohort spanning over a broader age range. With these simplifications, the measurement protocol should last less than 10 min, making it acceptable to be included in a screening workflow.

The limitations of this laboratory study are the low number of subjects and the absence of information about radiological mammographic density. Also, the optical power was not high enough to have a sufficient SNR along all the spectrum in transmittance measurements for some of the largest and/or densest breasts (Subject #7 and #10, in particular), where power attenuation is highest. Here our interest was focused on reflectance, and transmittance results were used as a reference. However, if necessary, the issue of low transmittance signal could be effectively removed with a multiple-detector probe^54^.

This study aimed at optimizing the setup and protocol in preparation for the future clinical trial. The hospital setting will then facilitate the test of breast density assessment through time domain diffuse optics on a larger population provided with recent mammograms, which will allow to correlate quantitative optical findings with radiological density information (*i.e.*, BI-RADS categories). The availability of a ground truth will then enable us to identify a minimal subset of wavelengths and measurement configurations to reliably estimate breast density. The ultimate goal is to make the diffuse optical assessment effective, short and cheap enough to become part of the routine exams in breast cancer screening programs.

### Supplementary Information

Supplementary Information.

## Data Availability

The dataset gathered, analyzed, and discussed in this study is available in the Zenodo repository, https://doi.org/10.5281/zenodo.10955509. For any additional information please contact the corresponding author.
