# Supplementary material for: In vivo optimization of the experimental conditions for the non-invasive optical assessment of breast density
Source: Sci Rep. 2024 Aug 19;14:19154. doi: 10.1038/s41598-024-70099-x (PMC11333589; doi:10.1038/s41598-024-70099-x)
Supplement: Supplementary file 1 — Supplementary Information. [file 41598_2024_70099_MOESM1_ESM.pdf]

Supplementary material: In vivo optimization of the experimental conditions for the non-invasive optical assessment of breast density.

Nicola Serra[1,], Rinaldo Cubeddu[1], Giulia Maffeis[1], Vamshi Damagatla[1], Antonio Pifferi[1,2] and Paola Taroni[1,2]*

[1]Dipartimento di Fisica, Politecnico di Milano, Piazza Leonardo da Vinci 32, 20133, Milano, Italy

[2]Istituto di Fotonica e Nanotecnologie, Consiglio Nazionale delle Ricerche, Piazza Leonardo da Vinci 32, 20133, Milano, Italy

*nicola.serra@polimi.it

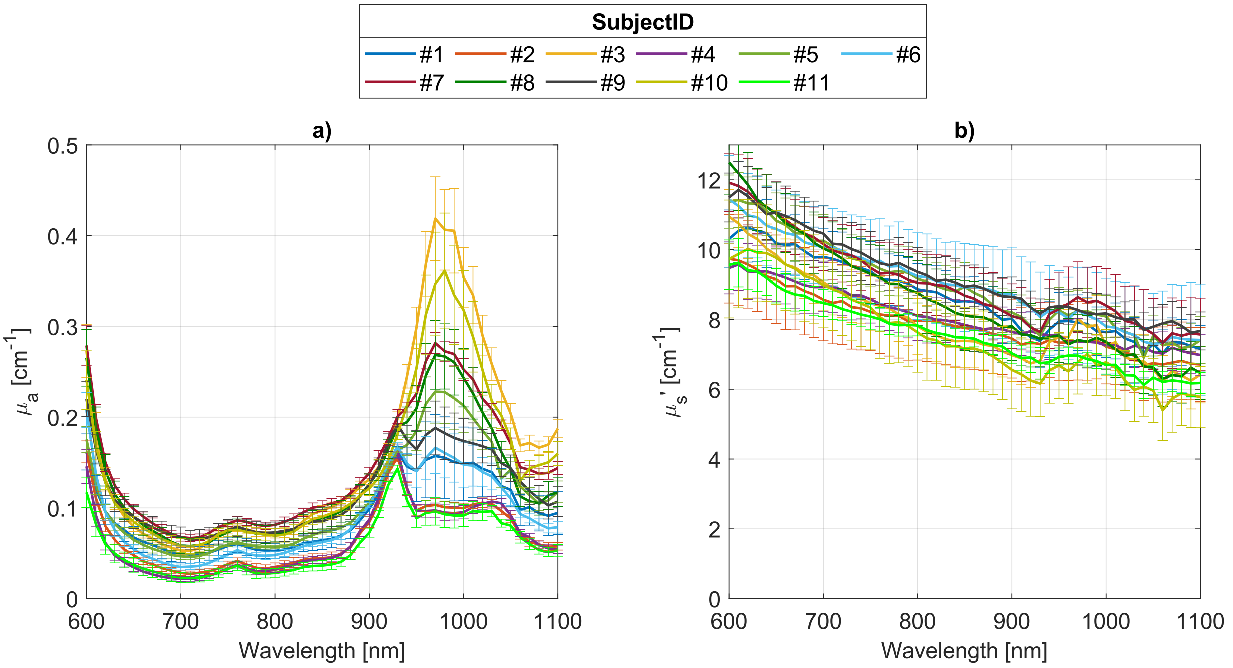

Supplementary Figure S1: Absorption (a) and reduced scattering (b) spectra of the 11 subjects (averaged over left and right breasts, and all measurement locations on the breast), in supine, reflectance configuration, at ρ = 2 cm.

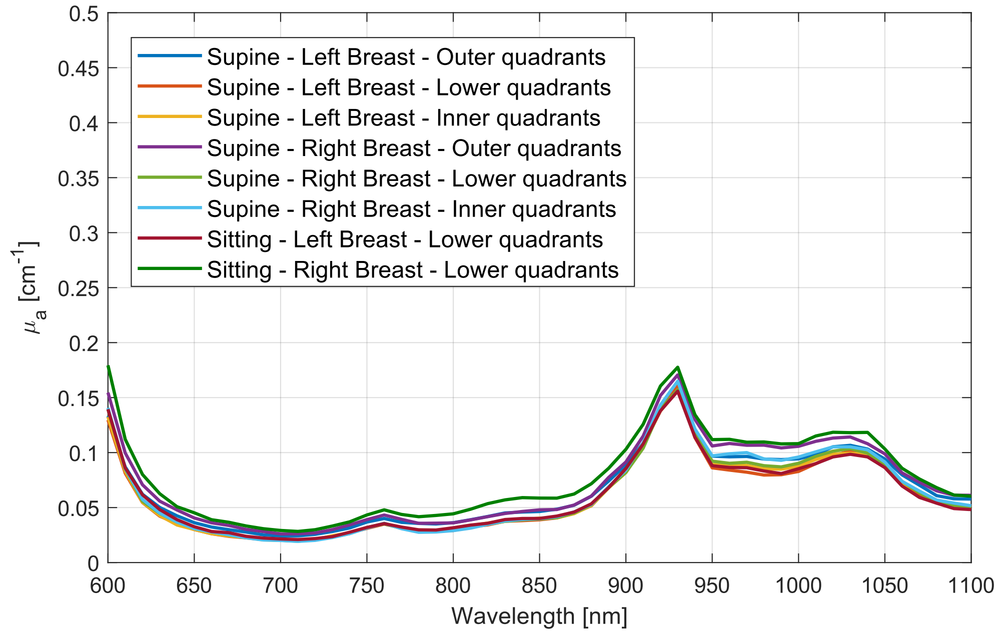

Supplementary Figure S2: Absorption spectra of typical adipose breast (subject #4), collected from the sitting subject (lower quadrants) and from the supine subject (inner, lower, and outer quadrants) at $\rho = 3$ cm.

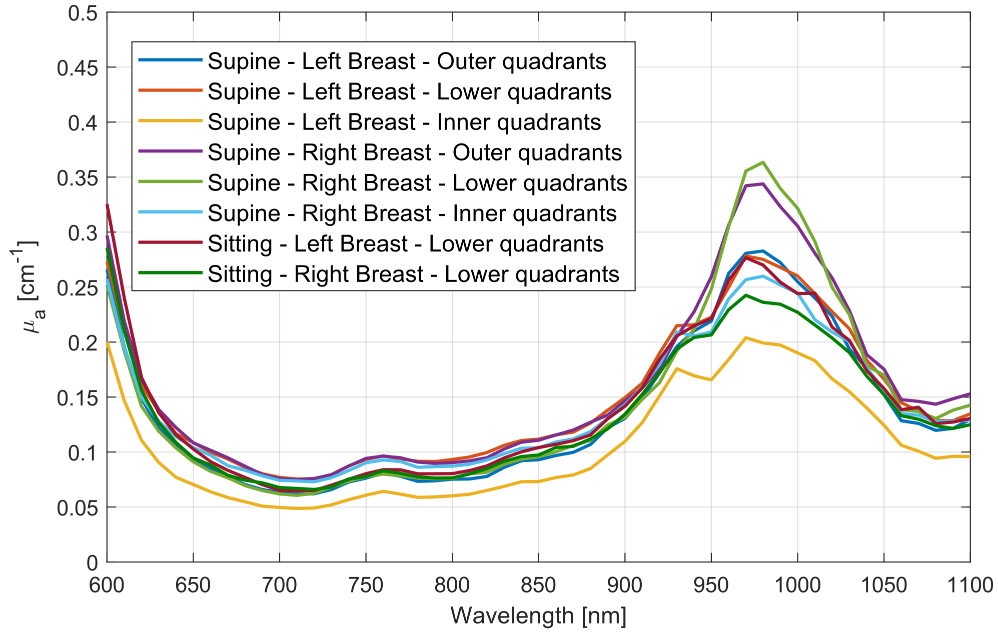

Supplementary Figure S3: Absorption spectra of typical mixed breast (subject #8), collected from the sitting subject (lower quadrants) and from the supine subject (inner, lower, and outer quadrants) at $\rho = 3$ cm.

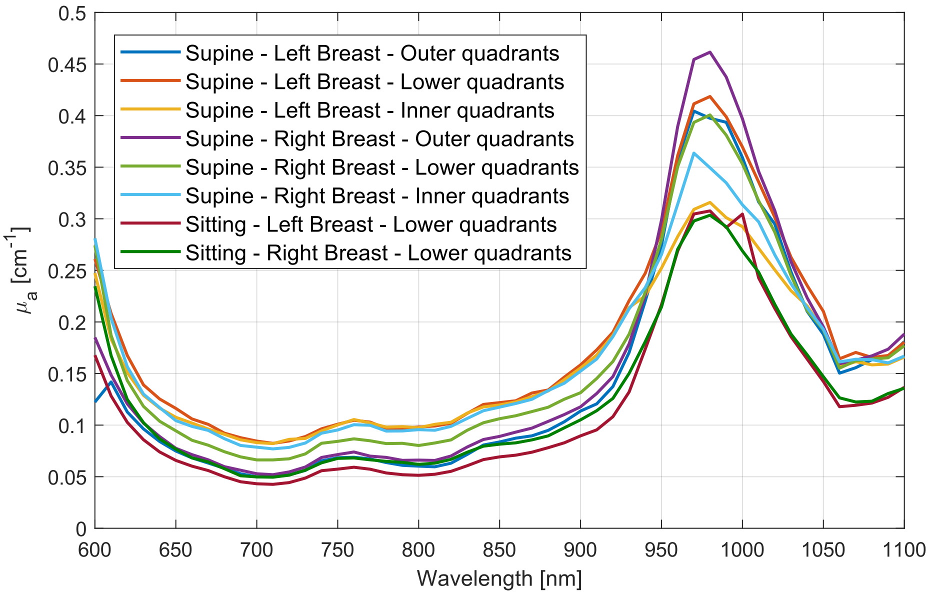

Supplementary Figure S4: Absorption spectra of typical fibro-glandular breast (subject #10), collected from the sitting subject (lower quadrants) and from the supine subject (inner, lower, and outer quadrants) at ρ = 3 cm.

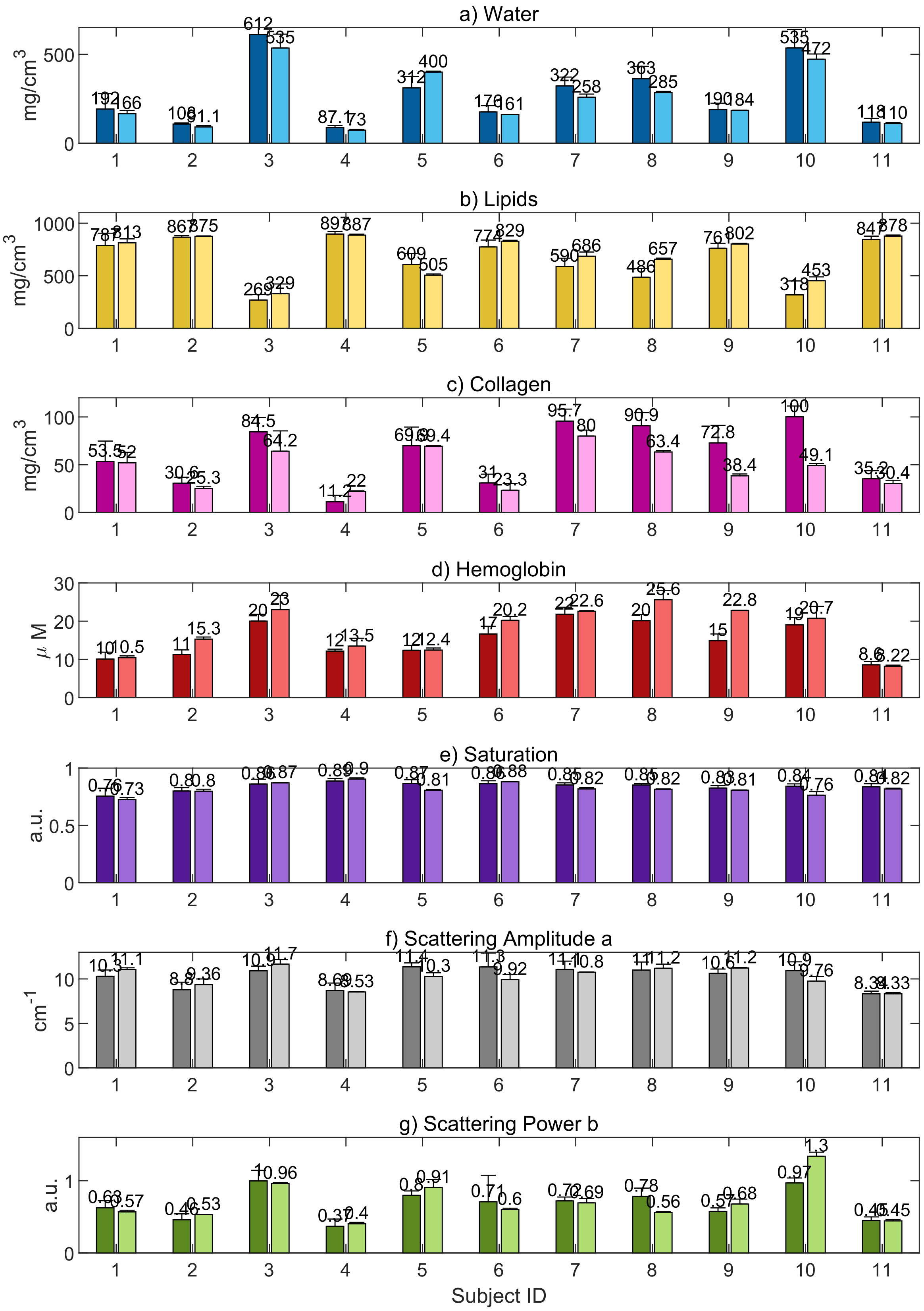

Supplementary Figure S5: Tissue component concentrations (a – c), blood (d, e) and scattering (f, g) parameters for the supine subject (dark colors), averaged over outer, lower, and inner quadrants, and for sitting subject (light colours), at ρ = 3cm, for all subjects.

	2 cm		3 cm	
	**r**	***p***	**r**	***p***
**Water**	-0.368	0.008**	-0.304	0.037**
**Lipid**	0.311	0.031**	0.292	0.047**
**Collagen**	-0.353	0.011**	-0.434	0.001**
**tHb**	-0.360	0.009**	-0.428	0.001**
**SO_2**	0.039	1.0	0.035	1.0
**a**	-0.575	3.1E-06**	-0.669	1.0E-08**
**b**	-0.362	0.009**	-0.447	0.001**

Table S1: Correlation coefficients (r) and p-values (p, adjusted for multiple testing) for the tissue constituent concentrations and scattering parameters measured in the supine protocol at ρ = 2 cm and ρ = 3 cm against the US breast thickness in the corresponding breast location.
